# A longitudinal cohort study observed increasing perfectionism and declining resilience, ambiguity tolerance and calling during medical school which is not explained by student personality

**DOI:** 10.1186/s12909-022-03850-5

**Published:** 2022-11-12

**Authors:** Diann S. Eley, Janni Leung, Kevin M. Cloninger

**Affiliations:** 1 Academy of Medical Education, Medical School, The University of Queensland, 288 Herston Road, Brisbane, QLD 4006 Australia; 2 National Centre for Youth Substance Use Research (NCYSUR), The University of Queensland, 17 Upland Road, St Lucia, QLD 4072 Australia; 3 The Anthropedia Foundation, St. Louis, MO USA

**Keywords:** Medical school culture, Hidden curriculum, Educational environment, Medical students, Perfectionism, Resilience, Ambiguity tolerance, Calling, Personality

## Abstract

**Background:**

The medical degree is a long and challenging program, not just academically, but regarding the expectations engrained in the culture of medical education. The recent proliferation of literature on the poor mental well-being among students suggests a dilemma that often lays the onus on students to improve their health. The link between personality and vulnerability to psychological distress is acknowledged. This longitudinal study looked at personality in 1^st^-year and changes in levels of certain psychological traits, as proxy indicators of well-being, in 4^th^-year. We aimed to determine to what extent changes in psychological traits over time may be attributed to personality.

**Methods:**

Medical students completed surveys at the start (1^st^-year: baseline) and finish (4^th^-year: follow-up) of their medical degree (*N* = 154). Temperament and character personality, Perfectionism-Concern over mistakes (CoM), Ambiguity Tolerance, Resilience, Calling to medicine, and demographic variables were measured. Paired t-tests compared changes in psychological traits from baseline to follow-up. Linear regression examined whether personality at baseline would predict levels of psychological traits at follow-up.

**Results:**

The temperament and character profile of the sample was as expected, and congruent with previous studies, which describe a mature personality. Over four years, levels of Perfectionism-CoM significantly increased, while Resilience, Ambiguity Tolerance and Calling to medicine decreased. Harm Avoidance, Persistence, Self-Directedness and Cooperativeness at baseline significantly predicted levels of these traits at follow-up, but effect sizes were weak. Correlations were in the expected direction and weak.

**Conclusions:**

Most commencing medical students, including this cohort, have mature personalities with an industrious temperament and an adaptable character. Yet over four years of medicine, Ambiguity Tolerance, Resilience and Calling declined while Perfectionism-CoM, already elevated at baseline, continued to increase to the final year. Of concern is the increased perfectionism that is strongly associated with poor mental health and psychological distress. The findings suggest a closer look at the entirety of the education environment and how its culture, including secondary school and the medical school admissions processes may influence these trends in students. As medical educators we should question why the pathway to medicine places such unhealthy pressure on students who aspire to be doctors.

**Supplementary Information:**

The online version contains supplementary material available at 10.1186/s12909-022-03850-5.

## Background

Almost exclusively, personality studies show that self-directedness or conscientiousness are the pivotal personality traits that predict performance in medical school and likewise general well-being [1–5]. Several studies have shown that certain profiles of personality traits are predictive of students who may be more vulnerable to stress [1, 2, 6–9]. Obviously, there is variation in students’ vulnerability to certain stressors, yet a growing concern is whether the medical education experience exacerbates this distress [10].

Studies show that a large proportion of enrolled medical students at the commencement of their degree had levels of burnout and depression and anxiety and stress higher than the general population [11, 12]. International studies with mixed cohorts also found this to be true. A meta-analysis of global prevalence of anxiety showed that one in three medical students have substantially higher levels of anxiety compared to the general population [13].

Other studies have shown that students begin medical school with a high level of perfectionism which could increase their vulnerability to stress and anxiety [14–17]. Perfectionism is a complex construct that deserves much attention especially among young people due to rapidly changing socio-cultural trends, including social media, promoting excessive comparison to, and competition with, others [18].

While it is possible that some personalities are better able to cope with elevated levels of perfectionism and associated stress, perfectionism was shown to be a mediator between personality and psychological distress [17]. That study suggests that while personality may play a part in how students cope with challenges in medical school, there are other factors to consider when fostering well-being during the four-year medical degree. This raises the point that it is important to consider whether the medical education environment or culture is partly culpable as a threat to mental health. Nevertheless, as background to this study, a brief description of personality and relevant psychological traits that may be indicative of well-being follows.

### Personality

This study employs Cloninger’s psychobiological theory of personality which proposes that the two interrelated domains of temperament and character interact in a non-linear system that is dynamic and regulates psychological functions [19]. An important contribution of the psychobiological approach is its link to the developmental stage of students and their educational environment. Temperament is the emotional core of personality. The traits are Novelty Seeking, Harm Avoidance, Reward Dependence, and Persistence. Character traits, Self-Directedness, Cooperativeness, and Self-Transcendence, reflect social-cultural learning and environmental influence. While character may develop across our lives, individual differences are as heritable as temperament [20, 21]. Each trait is multifaceted with high and low descriptors of each. (Additional file 1: Appendix 1) Previous research has consistently shown a temperament of low Harm Avoidance and high levels of Persistence with a character of high Self-Directedness and Cooperativeness as a mature personality and most often associated with well-being and satisfaction with life. Several studies show these relationships and aspects of well-being [4, 7, 9, 22].

### Selected psychological traits as proxy indicators of well-being

#### Ambiguity

Ambiguity is ubiquitous in medical practice [23]. Geller (et al. 2021) showed that tolerance for ambiguity is associated with levels of empathy. Research also shows that low levels of ambiguity tolerance are linked to higher rates of perceived stress [24] and burnout [25]. High levels are associated with people willing to work in rural or underserved areas [26, 27].

#### Perfectionism

There is abundant literature confirming perfectionism as a common characteristic of high achieving individuals and is prevalent in medical students and physicians [28]. The work by Hewitt, Flett and Frost over several decades unravels this complicated construct [29, 30]. While setting high standards and attention to detail have important benefits to health care, perfectionism also includes negative components. This study focussed on two components of Perfectionism. The first is ‘Concern over Mistakes’ (CoM) which represents a maladaptive form of perfectionism behaviours such as the inability to accept mistakes and setting self-defeating, unachievable goals [29, 31]. The other is ‘High Standards’ (HS) which represents positive achievement striving [32].

#### Resilience

Resilience has become so commonplace in discussions about well-being that we risk overlooking its genuine relevance to medical education. Although it can be argued that measuring resilience is not truly representative of one’s resilience potential, there is still value in recognising resilience as an important trait that can form part of our description of a psychologically mature personality. As a dynamic ‘process’ resilience manifests itself in response to life circumstances and one’s personality. Several studies have shown resilience as an indicator of a psychologically mature personality, which in turn is a strong predictor of the ability to cope with and bounce back from adversity [7, 33, 34].

#### Calling

Calling is associated with life meaning, motivation, self-efficacy, commitment and aspiring to goals and can have spiritual connotations [35]. The general concept of calling is often associated with health care professions and largely viewed as self-sacrificing and altruistic. Students in medicine, as compared to other health professions, are reported to be significantly higher in recognising a feeling of ‘Calling’ [36, 37].

### Current study

This paper reports on a four-year longitudinal study that investigated the personality and levels of selected psychological traits in a cohort of medical students from 1^st^-year (baseline) to 4^th^-year (follow-up) of their medical degree. We chose to monitor the above psychological traits based on prior research showing their inter-relationships with personality, and for their attributes that may be indicative of psychological distress i.e., proxy indicators of well-being. The aim of this study was to determine to what extent changes in psychological traits over time may be attributed to personality. We hypothesised that the influence of personality on changes in traits over the four-year degree would be typical and unlikely to be considered as the major influence in any changes we measure.

#### Research questions

We first describe the temperament and character personality profile of our sample and their accompanying levels of psychological traits at baseline and ask:

1. What is the association between personality and psychological traits at baseline?
2. How stable are psychological traits over time from baseline to follow-up?
3. Can personality at baseline predict levels of psychological traits at follow-up?

## Methods

### Design

This was a longitudinal study using a self-report questionnaire at two time-periods. The study was approved by the University of Queensland (UQ) Human Research Ethics Committee. All students provided written consent documented on the questionnaire.

### Participants and setting

The UQ medical program is a four-year graduate-entry MD degree and has an annual intake of approximately 480 students. Each yearly cohort is comprised of domestic Australians (65%) and approximately 35% international students, the majority of which represent Canada and the USA with the balance from Asia. All students complete the same curriculum.

All 1st-year students were invited to complete the online questionnaire during a scheduled activity within the first 2 months of the degree (baseline). Four years later, the same cohort were invited by email to complete the same survey minus the personality measurement within the last two months of their degree (follow-up). Both surveys asked for the student ID number. This allowed us to exactly match the respondents at both data collections. Only those students who responded at both time periods were included in the sample’s final analyses. We chose not to repeat the personality measure to reduce the survey burden on our students. The survey was accessed at both times via an online link (Survey Monkey©). All participants successfully completed the degree with no individual course failures.

### Measures

Demographic questions were age group (under or over 25 years), sex (male, female), student category (domestic, international), relationship status (married/partnered, single), and rural upbringing (yes, no), defined as spending most childhood years in a rural location.

#### Personality

Personality was measured by the Temperament and Character Inventory (TCIR-140) [19]. The 140 items are presented on a five-point Likert scale (1 = absolutely false to 5 = absolutely true). See Additional file 1: Appendix 1 for descriptors of each temperament and character trait.. Internal reliability of each trait was measured by Cronbach’s alpha and ranged from 0.70 to 0.92 for temperament and 0.88 to 0.89 for character.

#### Tolerance of ambiguity

The Multiple Stimulus Types Ambiguity Tolerance Scale-II (MSTAT-II) measured Ambiguity Tolerance (AT) [38]. The MSTAT-II uses a model that posits an individual’s perception of ambiguity is an orientation ranging from attraction to aversion toward stimuli that are uncertain, insoluble, or unfamiliar [38]. The 13 items use a Likert scale of 1 = definitely false to 5 = definitely true. Items are summed, with high scores indicating greater tolerance of ambiguity. Reliability alpha = 0.82.

#### Perfectionism

Two dimensions of the Frost Multidimensional Perfectionism Scale (FMPS) were used [29]. Concern over Mistakes (CoM) (8 items) represents a central concept of perfectionism that tends toward psychological distress. High Standards [HS] (5 items) reflects positive striving toward goals. Each item is presented as a five-point Likert scale from 1 = strongly disagree to 5 = strongly agree. Items are summed to derive scores for each subscale. A higher score indicates a higher level of perfectionism. Reliability alphas for each are 0.89 and 0.80 respectively.

#### Resilience

The Resilience Scale reflects the five core characteristics of resilience: perseverance, equanimity, meaningfulness, self-reliance, and existential aloneness [39]. It is a self-report measure of an individual’s ability to respond to adversity. The 14-item version uses a 7-point Likert-scale from 1 = Strongly Disagree to 7 = Strongly Agree. The single composite score represents high or low resilience. Reliability alpha = 0.91.

#### Calling

The Brief Calling Scale was used to assess the degree to which students see calling (to medicine) as relevant to their life and career [40]. The two question sub-scale indicates a “presence of calling” and is asked using a 5-point Likert scale of 1 = Not at all true of me, to 5 = Totally true of me. The scores are summed. A higher score indicates higher calling.

### Analysis

All analyses were conducted using SPSS 24 (SPSS Inc. Chicago, IL USA). Statistical significance was set at *p* < 0.05. Independent t-tests compared descriptive statistics of personality and psychological constructs by demographic variables at baseline. Pearson correlations, controlling for sex and age, provided the associations between the personality traits and measures of psychological constructs at baseline. Paired t-tests examined changes in psychological constructs from baseline to follow-up within the same individuals. Cohen’s *d* were calculated for the effect size. Linear regression analyses, adjusting for demographic characteristics, were conducted on levels of the psychological constructs at follow-up by personality at baseline. Standardized betas are reported.

## Results

### Sample description

#### Demographics

The final sample comprised 154 individuals who completed the surveys at baseline and follow-up. Overall, the response rates were 66% (i.e., 317/480 1^st^ year whole cohort) at the baseline data collection and 32% (i.e., 154/468 4^th^ year whole cohort) at follow-up data collection. The final sample follow-up rate was 49% (i.e., 154/317) of those students who completed both surveys. Table 1 provides the demographic description of the sample at baseline. Most students were under 25 years and single at both time periods, although 15 students were married or partnered by follow-up. A rural upbringing was reported by 25% of the sample. The proportions of students in our follow-up sample reflected the overall student cohort including student category (66% domestic), sex (53% female), and age ratio which did not change although students were four years older.

**Table 1:** Descriptive statistics at baseline: 1st-year MD (*N* = 154)

	Number	Valid %
Sex		
• Male	72	46.8
• Female	82	53.2
Age group		
• Under 25	96	62.3
• Over 25	58	37.7
Student Category		
• Domestic	101	65.6
• International	53	34.4
Relationship status		
• Married/Partnered	38	24.7
• Single	116	75.3
Rural background		
• Yes	38	24.8
• No	116	75.2

#### Baseline profile of temperament and character traits by demographics

Table 2 presents details of the levels of personality trait scores. The overall profile at baseline of temperament found low average levels of Novelty Seeking and Harm Avoidance, average Reward Dependence, and high Persistence. The character profile shows high Self-Directedness and Cooperativeness and average Self-Transcendence. Levels of personality traits by sex at baseline differed only in higher Self-Transcendence among females with a small effect size. Comparing student type showed significantly lower Harm Avoidance and higher Self-Directedness with strong effect sizes among international students compared to domestic students. Rural background students were higher in Novelty Seeking than non-rural background, and single compared to partnered students were higher in Self-Transcendence, both with medium to strong effect sizes. There was no difference in levels of any personality trait by age.

**Table 2:** Paired sample t-tests comparing temperament and character personality traits across baseline (1^st^year) and follow-up (4^th^year) by sex male (*n* = 72) and female (*n* = 82), and student type: domestic (*n* = 101) and international (*n* = 53)

	**Time 1: 1**^**st**^** year**						**Time 1: 1**^**st**^** year**					
	**Male**		**Female**				**Domestic**		**International**			
**Temperament and Character Personality**	M*	SD	M	SD	*p*	Cohen's *d*	M*	SD	M	SD	*p*	Cohen's *d*
Novelty Seeking	2.77	0.44	2.69	0.46	0.256	0.183	2.75	0.49	2.69	0.38	0.390	0.135
Harm Avoidance	2.65	0.69	2.82	0.74	0.145	-0.235	2.75	0.49	**2.48**	0.72	**0.001**	**0.555**
Reward Dependence	3.06	0.25	3.07	0.22	0.733	-0.055	3.07	0.25	3.07	0.22	0.845	0.033
Persistence	3.89	0.57	4.00	0.52	0.271	-0.178	3.91	0.52	4.02	0.57	0.232	-0.204
Self-Directedness	3.68	0.56	3.80	0.52	0.201	-0.207	3.66	0.50	**3.91**	0.59	**0.007**	**-0.462**
Cooperativeness	4.02	0.42	4.10	0.48	0.294	-0.169	4.05	0.41	4.10	0.54	0.523	-0.109
Self-Transcendence	2.55	0.67	**2.75**	0.74	0.010*	**-0.268**	2.61	0.67	2.80	0.78	0.208	-0.214

*M* Mean, *SD* Standard deviation

Whole sample (*N* = 154) Time1 Temperament and Character personality (TCIR-140) *scores: (Mean/SD): Novelty Seeking = 2.73/0.46; Harm Avoidance = 2.74/0.72; Reward Dependence = 3.07/0.24: Persistence = 3.95/0.54; Self-Directedness = 3.75/0.54; Cooperativeness = 4.06/0.45; Self-Transcendence = 2.66/0.71

^*^Mean personality score rankings: Very low (1.00- 1.50), low (1.51- 2.50), average (2.51- 3.50), high (3.51- 4.50) and very high (4.51- 5.00)

Time1 TCIR-140 by Rural Background showed that students reporting a rural background scored higher in Novelty Seeking (mean = 2.85; SD = 0.59) compared to those who did not have a rural upbringing (mean = 2.68; SD = 0.40), with a moderate effect size. (t = 1.89, 151; *p* < 0.051. Cohen’s *d* = 0.353)

Time1 TCIR-140 by Relationship Status showed that students who are single scored higher in Self-Transcendence (mean = 2.73; SD = 0.72) compared to those who are married or partnered (mean = 2.42; SD = 0.65), with a moderate effect size. (t = -2.50, 152; *p* < 0.015. Cohen’s *d* = -0.443)

Time 1 TCIR-140 by Age Group showed no difference

#### Baseline profile of psychological traits by demographics

Females reported a higher Calling to medicine at baseline compared to males. International students reported higher Ambiguity Tolerance, Resilience and Calling, with moderate effect sizes, compared to domestic classmates. There were no differences by any other demographic variable on levels of the psychological traits at baseline. Table 3 presents details.

**Table 3:** Means and standard deviations of Time 1 psychological traits by male (*n* = 72) and female (*n* = 82), and by student type: domestic (*n* = 101) and international (*n* = 53)

	Time 1: 1st year						Time 1: 1st year					
	**Male**		**Female**				**Domestic**		**International**			
	M	SD	M	SD	*p*	Cohen’s *d*	M	SD	M	SD	*p*	Cohen’s *d*
Ambiguity Tolerance	45.20	6.72	43.38	8.00	0.132	0.246	43.05	7.15	**46.49**	7.60	**0.006**	**0.470**
Concern over mistakes	23.70	6.11	24.36	6.43	0.513	0.105	23.79	6.04	24.55	6.72	0.474	0.119
High Standards	14.40	3.61	15.13	3.76	0.215	0.198	14.71	3.66	14.94	3.80	0.715	0.061
Resilience	81.56	10.43	83.45	9.43	0.239	0.190	81.12	8.98	**85.31**	11.09	**0.012**	**0.429**
Calling to medicine	7.19	2.08	**7.95**	2.03	**0.024**	**0.369**	3.65	0.95	**4.07**	1.14	**0.017**	**0.411**

Mean score ranges:

• Tolerance of Ambiguity score range: M = 41.4 (*SD* = 7.5)—M = 44.0 (SD = 7.18)

• Perfectionism: Concern over Mistakes score range: 8–22. Cut-off score of 19 or higher indicates distress due to perfectionism

• Perfectionism: High Standards score range: Range 5–25

• Resilience score range: Very low = 14–56; Low = 57–64; Moderate low = 65–73; Moderate high = 74–81; High = 82–90; Very high = 91–98

• Calling score range: 2 – 10

##### Question 1: What is the association between personality and psychological traits at baseline?

Table 4 presents the correlation matrix. Ambiguity Tolerance and Resilience show strong negative correlations with Harm Avoidance, in contrast to their strong positive associations with Persistence, Self-Directedness and Cooperativeness. Resilience is strongly positive with Ambiguity Tolerance and Calling but negative with Perfectionism-CoM. The correlations between Perfectionism-CoM with the personality traits were weak and non-significant.

**Table 4:** Correlation matrix between temperament and character personality and psychological traits at baseline

	Novelty Seeking	Harm Avoidance	Reward Dependence	Persistence	Self-Directedness	Cooperativeness	Self-Transcend ence	Ambiguity Tolerance	Concern over Mistakes	High Standards	Resilience
Ambiguity Tolerance	0.121	**-.561**^**a**^	0.116	**.381**^**a**^	**.407**^**a**^	**.417**^**a**^	0.112	–			
Concern over Mistakes	-0.134	.272^a^	-0.034	0.035	-.262^a^	-.249^a^	-.176^b^	-.264^a^	–		
High Standards	-0.080	-0.122	-0.136	**.625**^**a**^	.197^b^	0.099	0.147	.166^b^	.269^a^	–	
Resilience	-0.001	**-.610**^**a**^	0.023	**.576**^**a**^	**.680**^**a**^	**.371**^**a**^	.166^b^	**.411**^**a**^	**-.319**^**a**^	.235^a^	–
Calling to Medicine	-0.156	-.184^b^	0.025	**.423**^**a**^	.276^a^	.231^a^	.250^a^	.271^a^	0.016	**.337**^**a**^	**.305**^**a**^

Coefficients over 0.3 are in bold

^a^Correlation is significant at the 0.01 level (2-tailed)

^b^Correlation is significant at the 0.05 level (2-tailed)

##### Question 2: How stable are psychological traits over time?

Table 5 shows the comparison between baseline and follow-up levels of the psychological traits. Perfectionism-CoM significantly increased, while Resilience and Calling decreased significantly over time. Effect sizes were small.

**Table 5:** Paired sample t-tests comparing the whole cohort (*N* = 154) psychological traits across baseline (1^st^year) and follow-up (4^th^year)

Psychological traits	Time	Mean	Std. Dev	Std. Error	*p*	Cohen’s *d*
Ambiguity Tolerance	Baseline	44.23	7.46	0.60		
	Follow-up	43.48	7.37	0.59	0.141	-0.119
Concern over mistakes	Baseline	24.00	6.27	0.50		
	Follow-up	**25.58**	6.133	0.49	**0.002**	**0.254**
High Standards	Baseline	14.79	3.69	0.29		
	Follow-up	14.83	3.57	0.28	0.880	0.012
Resilience	Baseline	82.57	9.92	0.80		
	Follow-up	**80.42**	9.96	0.80	**0.001**	**-0.278**
Calling to medicine	Baseline	7.60	2.08	0.16		
	Follow-up	**6.88**	2.47	0.20	** < 0.001**	**-0.333**

There were some significant changes over time by demographic variables. Females were lower in Ambiguity Tolerance and higher in Calling to medicine compared to males with moderate effect sizes. International students were higher in Ambiguity Tolerance at baseline but by follow-up levels were nearly equal. Resilience at baseline was higher among international students but at follow-up, while they remained higher in Resilience, levels had decreased to non-significance. International students were higher in Calling at both time points compared to Domestics. All effect sizes were moderate. See Additional file 1: Appendix 2.

##### Question 3: Can personality at baseline predict levels of psychological traits at follow-up?

The linear regression model (Table 6) showed only weak associations. Harm Avoidance predicted lower Ambiguity Tolerance. Cooperativeness predicted higher Ambiguity Tolerance and lower Perfectionism-CoM. Persistence predicted higher Perfectionism-HS, Resilience, and Calling. Self-Directedness predicted higher Resilience.

**Table 6:** Linear regression^a^ on psychological traits at follow-up (4^th^year) by TCIR-140 personality at baseline (1^st^year)

	**Psychological traits outcomes at follow-up**									
**Baseline Temperament and Character Personality**	Ambiguity Tolerance		Concern Mistakes		High Standards		Resilience		Calling	
	*β*	*p*	*β*	*p*	*β*	*p*	*β*	*p*	*β*	*p*
Novelty Seeking	0.11	0.174	-0.09	0.374	-0.06	0.480	0.02	0.833	-0.01	0.901
Harm Avoidance	**-0.43**	** < 0.001**	-0.05	0.664	-0.07	0.501	-0.08	0.436	-0.20	0.062
Reward Dependence	-0.11	0.146	0.13	0.158	-0.07	0.367	-0.07	0.369	-0.06	0.500
Persistence	0.15	0.075	0.15	0.146	**0.49**	** < 0.001**	**0.32**	** < 0.001**	**0.26**	**0.004**
Self-Directedness	-0.10	0.311	-0.16	0.187	-0.01	0.921	**0.32**	**0.001**	0.04	0.711
Cooperativeness	**0.30**	** < 0.001**	**-0.28**	**0.004**	0.06	0.482	0.11	0.144	0.04	0.621
Self-Transcendence	**-0.25**	**0.002**	-0.03	0.768	-0.02	0.842	-0.04	0.613	0.14	0.099

^a^Regression model adjusted for sex, age group, student type, marital status, and rural background (see Additional file 1: Appendix 3 for coefficients)

Independent variables: personality traits at baseline Time 1

Dependent variables: Ambiguity Tolerance (AT), Perfectionism Concern over Mistakes (CoM) and High Standards (HS), Resilience and Calling to Medicine (Calling) at follow-up Time 2

A linear regression on psychological traits at follow-up (4^th^year) by demographic variables at baseline (1^st^year) showed weak associations between Relationship Status with Ambiguity Tolerance and Rural Background with Calling to medicine (Additional file 1: Appendix 3).

## Discussion

This study investigated temperament and character personality and selected psychological traits as indicators of well-being, in a sample of medical students in 1st-year (baseline) and 4th-year (follow-up) of their medical degree. The aim was to determine to what extent changes in psychological traits over time may be attributed to personality. We hypothesised that the influence of personality on changes in traits over the four-year degree would be typical and unlikely to be the major influence in any changes we measured. The findings support our hypothesis. While personality correlated strongly with some traits in the expected direction and predicted changes in some of the traits—associations were weak. Furthermore, the overall personality profile of the sample was as expected, and congruent with previous studies, which describe a mature personality (detail below). Therefore, we posit that other factors may be contributing to influence these findings, the implications of which will also be discussed below.

### Describing our sample’s personality profile

The first question asked, what is the association between personality and psychological traits at baseline? The temperament and character personality profile measured at baseline is congruent with previous research [7–9] which portrays a mature personality distinguished by a temperament of low-average Harm Avoidance and high Persistence, complemented by a character high in Self-Directedness and Cooperativeness. This profile also corresponds with studies using the five-factor model of personality [41] showing similar combinations of Conscientiousness, Extraversion and Agreeableness found to be predictive of academic performance and overall success and well-being in medical school [1, 3, 5].

The temperament trait Harm Avoidance is a measure of anxiety proneness and predicted lower Ambiguity Tolerance, which is characterised as perceiving threat from situations that are inexplicable or complicated [23]. With temperament we often label ourselves by descriptors that feel innate and intense (i.e., either/or extremes) such as worrier and tense, or confident and optimistic. Although describing Harm Avoidance is not that clear-cut, an inability to accept uncertainty (i.e., high Harm Avoidance), implies low Ambiguity Tolerance.

The temperament trait Persistence represents industriousness and diligence despite obstacles and predicted levels of Perfectionism-HS, Resilience and Calling to medicine. Research consistently shows that medical students score very high on Persistence which may be responsible for its strong positive association with Calling and Perfectionism-HS [32, 35]. Both require a passion to achieve and speaks to the many years of dedication maintaining high standards to reach medical school, which for many students has been a strong desire or “calling” for several years. Resilience in particular is bolstered and maintained by high levels of Persistence and Self-Directedness.

The character trait Self-Directedness represents conscientiousness, responsibility, and self-acceptance, and is consistently high in medical students [5, 8]. Self-Directedness is shown to be the most beneficial trait contributing to life satisfaction and overall well-being [42]. Furthermore, the combination of high Self-Directedness and Persistence provides the strongest positive associations with Resilience [4, 7].

The character trait Cooperativeness predicted higher levels of and is strongly correlated with Ambiguity Tolerance. Cooperativeness represents patience, empathy, and agreeableness, which are key aspects of being tolerant of uncertainty. The association between ambiguity tolerance and Cooperativeness is manifested in empathy [23]. In contrast, Cooperativeness also predicted lower levels of Perfectionism-CoM. Perfectionism-CoM is the psychological trait of most concern in this study and the biggest threat to our students’ vulnerability to psychological distress. An important component of Cooperativeness is the degree to which a person is generally agreeable and empathic to others with a willingness to support without any self-serving purpose or self-centred nature [42]. Cooperativeness offers some protective element to becoming too focussed on self and the desire to be “perfect” – when perfection is unattainable and self-defeating.

### Demographic associations with personality

The only demographic variable which was significant in the regression model was rural background which predicted higher levels of Calling to medicine. This finding has anecdotal support through stories of students from rural or underserved backgrounds that overcome hardships to attain a place in medicine. A “calling” aptly describes this circumstance. Otherwise, there were few significant differences in personality by demographics. While we found the usual trends with females higher than males in Harm Avoidance, Reward Dependence and Cooperativeness, these were not significant. Similar to a recent study, our international students scored significantly lower in Harm Avoidance and higher in Self-Directedness compared to their domestic classmates [43]. The combination of these two traits, while not decisive, may suggest more maturity in international students. This observation may reflect an influence due to a different educational background or a personal quality indicative of commitment to achieve their aims.

### Personality is associated with levels of psychological traits in 1st-year.

Our findings confirm previously seen trends showing strong negative associations between Harm Avoidance with Resilience and Ambiguity Tolerance. Conversely, Ambiguity Tolerance and Resilience have strong positive associations with Persistence, Self-Directedness and Cooperativeness. These associations are important for medical students to consider early in their medical training. Improving individuals understanding of their personality, especially certain combinations of traits that may influence reactions or subsequent behaviour, can help develop self-awareness and recognise their personal strengths and weaknesses [9].

### Levels of psychological traits changed over time

The second question asked how stable are psychological traits over time? The traits we measured were chosen as proxy indicators of well-being because of their relationship with temperament and character personality profiles [8, 9]. Levels of all traits decreased over time except for Perfectionism-CoM which started high at baseline and continued to increase, and Perfectionism-HS which did not change. The latter may seem unsurprising suggesting that high achievers do not falter in their level of striving for personal goals and achievement. While having high standards can drive achievement, it may also feed the more negative aspect of perfectionism that can become maladaptive. This negative aspect of perfectionism is reflected in Perfectionism-Concern over Mistakes (CoM) as evaluative concern [31]. High levels of Perfectionism-CoM are consistently shown to be a contributor to psychological distress, which includes negative feelings, excessive self-criticism, indecision, anxiety, and fear of failure [9, 16, 17, 31, 32, 44, 45]. A further increase in Perfectionism-CoM over time indicates that our students’ vulnerability to psychological distress may also be increasing. A strong sense of resilience and calling could add meaning to their degree and help overcome such negativity. Yet we found that levels of Resilience and Calling both decreased over time by Year-4 follow-up.

### Personality can predict levels of psychological traits over time.

The last question asked if personality at baseline predicted levels of psychological traits at follow-up? There is considerable literature that shows personality can predict changes in levels of other traits and later outcomes [1, 4, 7, 22]. We measured personality at baseline to demonstrate the consistent finding that most students entering medicine have a mature personality that should equip them to cope with challenges of a demanding degree. While personality did predict levels of traits in final year, the associations were weak. The correlation matrix of the psychological traits with personality showed the expected relationships based on previous studies [9, 27], except that Perfectionism-CoM did not correlate even moderately with temperament or character. Furthermore, baseline levels of the psychological traits (Ambiguity Tolerance, Resilience and Calling) were within the high/normal range. Only Perfectionism-CoM was elevated at to an abnormal level which indicates risk for psychological distress. To note, it is important to consider that most students have already spent many years of education competing for the best grades and the highest achievements to gain a place in medicine. This may in part explain the elevated levels of Perfectionism-CoM in 1^st^-year medicine, it also suggests that some students come into medicine with a higher risk for distress.

### Limitations

This study has limitations including its ability to generalise the findings for reasons common to observational studies; nor does it inform causality. A control or a comparator group would be necessary to indicate any change over time was due to the medical school experience, as would a measure of students’ perception of their culture. Research exploring students’ perception of their educational environment across their degree is necessary and could help identify negative aspects of the education experience and understand how some aspects have become inculcated into the culture of medicine. Perfectionism is a multifaceted construct and only two measures were used for this study which may limit our conclusions.

The sample comes from one institution and one cohort that may not be representative of other medical student populations. This was a longitudinal study that required a follow-up data collection after four years. There are recognised challenges to students engaging in surveys, yet we achieved a suitable number of students retained at follow-up, and their demographics were representative of the whole cohort in age, domestic and international status, and sex. It is important to note as a possible limitation, that this sample self-selected to participate, and all measures are self-report.

Our study did not look at academic performance except to note that all students satisfactorily completed the degree with no course failures over the four years. It could be assumed that this cohort of students, as part of a much larger class, are exemplar in their personalities i.e., highly cooperative, and self-directed, with a greater interest in this research.

## Conclusions

Given the copious literature about the decline in medical student well-being, it might seem surprising if levels of some traits didn’t deteriorate over time. However, the increase in Perfectionism-CoM may be especially concerning as 1st-year students were already at levels that may cause or exacerbate distress. We should acknowledge that many students begin medical school with what Slavin [46, 47] refers to as “problematic mindsets”, driven by increasing comparison to and competitiveness with others, and fuelled in part by social trends [18].

However, significant consideration must point to the years of competitiveness and pressure placed on students with the goal of admission to medicine. Studies have examined the role of parental expectations and pressure on their children to excel at school and college and found positive correlations with the negative maladaptive aspects of perfectionism which are increasing over time [18, 48]. Unfortunately, in first year medicine we are seeing the effects of a prevalence of psychological distress developed during secondary and tertiary education [49]. The concern for medical educators is that these students are entering an environment that may introduce and or exacerbate this distress, possibly to the point of developing more serious psychological problems [46].

We continue to see that the students we choose to admit to medical school, in general, have personalities that would be expected of intelligent high achieving individuals training for, and working as health professionals [8, 9]. Yet we also see increasing burnout, depression, and suicidal ideation prominent throughout the continuum of medical education and training [50, 51].

We propose that the culture and expectations of medical school is partly culpable as a threat to developing or exacerbating negative perfectionistic attitudes in students that undermine their potential for well-being. To be clear, by culture and expectations, we imply the gamut of education and the years of preparation involved to gain admission to medical school. This includes the culture of competitiveness and comparison that is evident as early as middle school through to university and exacerbated by unrealistic expectations and criticism from parents and largely unavoidable social media. [18, 48]. Medical education should be an opportunity for learners to strengthen their personalities and resilience through an educational environment that is challenging but supportive and nurturing, where expectations and boundaries are clear and enforced. Medical educators should question if the intense competitive pathway into medicine could be less so for students who aspire to become a doctor.

## Supplementary Information

**Additional file 1: Appendix 1.** High and low descriptors for each temperament and character personality trait. **Appendix 2.** Paired sample t-tests comparing the psychological traits across baseline (1^st^year) and follow-up (4^th^year) by sex male (*n*=72) and female (*n*=82), and student type: domestic (*n*=101) and international (*n*=53). **Appendix 3.** Linear regression on psychological traits at follow-up (4^th^year) by demographic variables at baseline (1^st^year).

## Data Availability

The datasets used and/or analysed during the current study are available from the corresponding author on reasonable request.
