# Supplementary material for: A longitudinal cohort study observed increasing perfectionism and declining resilience, ambiguity tolerance and calling during medical school which is not explained by student personality
Source: BMC Med Educ. 2022 Nov 12;22:784. doi: 10.1186/s12909-022-03850-5 (PMC9655808; doi:10.1186/s12909-022-03850-5)
Supplement: Supplementary file 1 — Additional file 1: Appendix 1. High and low descriptors for each temperament and character personality trait. Appendix 2. Paired sample t-tests comparing the psychological traits across baseline (1styear) and follow-up (4thyear) by sex male (n=72) and female (n=82), and student type: domestic (n=101) and international (n=53). Appendix 3. Linear regression on psychological traits at follow-up (4thyear) by demographic variables at baseline (1styear). [file 12909_2022_3850_MOESM1_ESM.docx]

**Appendix 1**: High and low descriptors for each temperament and character personality trait

| **Temperament traits** | ***Represents………*** | **LOW SCORES** |  | **HIGH SCORES** |
| --- | --- | --- | --- | --- |
| Novelty Seeking  [NS] | *Exploratory activity in response to novelty* | *Orderly, reflective, reserved* |  | *Exploratory, curious,*  *seeks challenge* |
| Harm Avoidance  [HA] | *Worry in anticipation of problems* | *Confident, accepting of uncertainty & risk* |  | *Anxious, uncomfortable*  *with accepting risk* |
| Reward Dependence [RD] | *Dependence on approval of others* | *Not influenced by others, objective, insensitive* |  | *Needs to please, warm, attached, sociable* |
| Persistence  [PS] | *Industriousness despite obstacles* | *Quitting, underachiever, erratic, unambitious* |  | *Ambitious, hard worker, diligent, perfectionist* |
| **Character**  **traits** | ***Represents………*** | **LOW SCORES** |  | **HIGH SCORES** |
| Self-Directedness [SD] | *Responsibility, goal orientated & self-confidence* | *Blaming, ineffective, unreliable, irresponsible* |  | *Conscientious, self-accepted, reliable,* |
| Cooperativeness  [CO] | *Tolerance, empathy & cooperativeness* | *Intolerant, critical opportunistic, unhelpful* |  | *Tolerant, agreeable, constructive, empathic* |
| Self-Transcendence [ST] | *View of self in relation to universe* | *Impatient, proud, materialistic, practical* |  | *Patient, humble, spiritual, creative, compassionate* |
| *Adapted from Eley et al. 2017 | | | | |

**Appendix 2**: Paired sample t-tests comparing the psychological traits across baseline (1^st^year) and follow-up (4^th^year) by sex male (n=72) and female (n=82), and student type: domestic (n=101) and international (n=53).

|  |  | **Male** | | **Female** | |  | |  | **Domestic** | | **International** | |  | |
| --- | --- | --- | --- | --- | --- | --- | --- | --- | --- | --- | --- | --- | --- | --- |
| **Traits** | **Time** | M | SD | M | SD | *p* | Cohen's *d* | **Time** | M | SD | M | SD | *p* | Cohen's *d* |
| **Ambiguity Tolerance** | T1 | 45.20 | 6.72 | 43.38 | 8.00 | 0.132 | 0.369 | T1 | 43.05 | 7.15 | **46.49** | 7.60 | **0.006** | 0.470 |
|  | T2 | 44.98 | 6.96 | **42.12** | 7.51 | **0.015** |  | T2 | 43.54 | 7.70 | 43.35 | 6.77 | 0.882 |  |
| **Concern over mistakes** | T1 | 23.70 | 6.11 | 24.36 | 6.43 | 0.513 | <0.2 | T1 | 23.79 | 6.04 | 24.55 | 6.72 | 0.474 | <0.2 |
|  | T2 | 25.16 | 6.22 | 25. 96 | 6.05 | 0.422 |  | T2 | 25.07 | 6.18 | 26.57 | 5.97 | 0.151 |  |
| **High Standards** | T1 | 14.40 | 3.61 | 15.13 | 3.76 | 0.215 | <0.2 | T1 | 14.71 | 3.66 | 14.94 | 3.80 | 0.715 | <0.2 |
|  | T2 | 14.79 | 3.42 | 14.86 | 3.73 | 0.904 |  | T2 | 14.64 | 3.67 | 15.19 | 3.38 | 0.359 |  |
| **Resilience** | T1 | 81.56 | 10.43 | 83.45 | 9.43 | 0.239 | <0.2 | T1 | 81.12 | 8.98 | **85.31** | 11.09 | **0.012** | 0.429 |
|  | T2 | 79.13 | 10.51 | 81.58 | 9.36 | 0.129 |  | T2 | 79.36 | 10.02 | 82.43 | 9.63 | 0.067 |  |
| **Calling to medicine** | T1 | 7.19 | 2.08 | **7.95** | 2.03 | **0.024** | 0.395 | T1 | 3.65 | 0.95 | **4.07** | 1.14 | **0.017** | 0.411 0.474 |
|  | T2 | 6.51 | 2.50 | 7.22 | 2.42 | 0.074 |  | T2 | 6.49 | 2.44 | **7.64** | 2.37 | **0.006** |  |

**Appendix 3:** Linear regression on psychological traits at follow-up (4^th^year) by demographic variables at baseline (1^st^year).

|  |  | **Psychological trait outcomes at follow-up** | | | | | | | | | |
| --- | --- | --- | --- | --- | --- | --- | --- | --- | --- | --- | --- |
|  |  | Ambiguity Tolerance | | Concern over Mistakes | | High Standards | | Resilience | | Calling | |
|  | | *β* | *p* | *β* | *p* | *β* | *p* | *β* | *p* | *β* | *p* |
| **Baseline demographics** | |  |  |  |  |  |  |  |  |  |  |
|  | Sex | -0.09 | 0.235 | 0.08 | 0.347 | -0.02 | 0.789 | 0.08 | 0.233 | 0.11 | 0.139 |
|  | Age | -0.06 | 0.433 | 0.03 | 0.684 | 0.03 | 0.713 | 0.04 | 0.597 | -0.10 | 0.198 |
|  | Student type | -0.09 | 0.254 | 0.03 | 0.711 | -0.01 | 0.946 | 0.02 | 0.834 | 0.06 | 0.435 |
|  | Relationship status | **-0.15** | **0.040** | 0.11 | 0.228 | 0.07 | 0.348 | -0.02 | 0.732 | -0.02 | 0.797 |
|  | Rural background | 0.11 | 0.116 | 0.00 | 0.974 | 0.03 | 0.658 | 0.05 | 0.508 | **0.17** | **0.024** |

*Independent variables: Demographics at Time 1: Sex: male vs female; Age: under 25 vs over 25; Student type: domestic vs international; Relationship status: single vs married/partnered; Rural Background: rural vs not rural
